# Oral health, dietary variety, homebound status, and intrinsic capacity among community-dwelling older adults

**DOI:** 10.1016/j.jnha.2026.100880

**Published:** 2026-05-19

**Authors:** Takamasa Komiyama, Takashi Ohi, Eiko Sato, Moe Sato, Yoshinori Hattori

**Affiliations:** a Division of Aging and Geriatric Dentistry, Department of Rehabilitation Dentistry, Tohoku University Graduate School of Dentistry, Sendai, Miyagi, Japan; b Japanese Red Cross Ishinomaki Hospital, Ishinomaki, Miyagi, Japan

**Keywords:** Oral health, Oral frailty, Intrinsic capacity, Older adults, Dietary diversity, Homebound

## Abstract

**Purpose:**

This study aimed to examine the association between oral health indicators and intrinsic capacity among community-dwelling older adults, and to determine whether dietary variety or homebound status moderated this association.

**Methods:**

The study included 692 community-dwelling Japanese older adults. Oral health indicators included mastication, oral motor function, salivation, swallowing, oral frailty (integrating these four components) based on the latest international e-Delphi study and tongue pressure. All indicators were assessed using oral examinations or validated questionnaires. Intrinsic capacity deficits were assessed across five domains: vitality, psychological, cognitive, locomotive, and sensory. Intrinsic capacity was operationalized as the number of deficits across the five domains. Data on dietary variety and homebound status were also collected. The covariates included age, sex, education, smoking status, drinking status, and comorbidities. Modified Poisson regression models were used to estimate rate ratios (RRs) and 95% confidence intervals (CIs) for the associations between oral health indicators and intrinsic capacity deficits. Interaction terms between oral frailty and dietary variety or homebound status were also included.

**Results:**

The prevalence of sensory, locomotive, psychological, cognitive, and vitality deficits was 41.2%, 7.4%, 11.9%, 22.0%, and 17.3%, respectively. In the multivariate model, individuals having more oral frailty deficits (RR, 1.16; 95% CI, 1.08–1.24), decreased tongue pressure (RR, 1.20, 95% CI, 1.04–1.39), lower oral motor function (RR, 1.22; 95% CI, 1.05–1.42), declined swallowing function (RR, 1.25; 95% CI, 1.08–1.46), and oral dryness (RR, 1.32; 95% CI, 1.15–1.53) were more likely to have intrinsic capacity deficits. No significant interactions were observed between oral frailty and dietary variety, or between oral frailty and homebound status, on intrinsic capacity.

**Conclusion:**

This study showed that cumulative declines in oral health were associated with intrinsic capacity deficits in community-dwelling older adults; moreover, effect modification by dietary variety or homebound status was not identified. Integrated dental services may help maintain intrinsic capacity in older adults; however, longitudinal studies are warranted to better understand this association.

## 1 Introduction

The number of older adults is increasing worldwide and is estimated to reach 2.1 billion by 2050, more than double the number in 2019 (1 billion) [1]. The United Nations has designated 2021–2030 as the Decade of Healthy Ageing. According to the World Report on Ageing and Health (2015), healthy ageing is defined as *“the process of developing and maintaining the functional ability that enables well-being in older age”* [2]. In this framework, intrinsic capacity is defined as a composite of all physical and mental functions, whereas functional ability comprises the intrinsic capacity of the individual, relevant environmental characteristics, and interactions between the individual and these characteristics [2]. Furthermore, it has been suggested that oral health should be integrated into assessments together with physical and psychological evaluations (e.g., vitality, locomotion, cognition, and psychological), given its important role in maintaining nutritional status and functional capacity in later life.

Accumulating evidence suggests that oral health plays a significant role in healthy aging later in life, particularly in relation to frailty, sarcopenia, and functional disability [[3], [4], [5]]. Regarding oral health indicators, tooth loss, periodontal disease, dental health behaviors, dental prostheses, and oral function are associated with adverse health outcomes [[5], [6], [7], [8], [9]]. With respect to intrinsic capacity, oral health may influence vitality, locomotion, cognition, and psychological capacity through multiple pathways, including dietary variety and nutritional status due to impaired masticatory function, systemic inflammation due to periodontitis, and social withdrawal due to aesthetic and functional concerns (e.g. tooth loss) and dental pain [[10], [11], [12]].

Regarding the association between oral health and intrinsic capacity, tooth loss, periodontal disease, and self-reported oral health were associated with intrinsic capacity based on NHANES data [[13], [14], [15]]. Another report from a frailty clinic examined whether intrinsic capacity played a significant role in maintaining oral function, where oral health assessment was conducted by a physical therapist [16]. However, to our knowledge, no studies have examined the association between comprehensive oral functional decline and intrinsic capacity deficits among community-dwelling older adults nor explored the potential heterogeneity in this association. This study aimed to (1) examine the association between oral functional indicators and intrinsic capacity among community-dwelling Japanese older adults and (2) examine whether dietary variety or homebound status moderated this association. We hypothesized that declined oral function is significantly associated with intrinsic capacity and that both dietary variety and homebound status can moderate this association.

## 2 Material and methods

### 2.1 Study design

The participants, who were invited by municipal authorities from selected districts, were community-dwelling older adults who attended municipal health checkups conducted between May 2024 and October 2025 across 29 districts in 11 municipalities in Akita and Miyagi Prefectures, Japan. Supplemental Fig. S1 shows a flow diagram of the study participants. A total of 834 participants underwent the initial examination. Among them, three participants were excluded because they were aged less than 65 years, 49 because of lack of informed consent, one because of missing oral health information, and 89 because of missing data on at least one domain of intrinsic capacity. Finally, 692 participants were included in the final analysis. This study was approved by the Ethics Committee of Tohoku University Graduate School of Dentistry (Approval No. 35745). Data were obtained from participants who provided written informed consent.

### 2.2 Oral health assessment

The oral health assessment consisted of objective examinations performed by dental professionals such as dentists and dental hygienists and an oral health-related questionnaire. Prior to data collection, the examiners standardized the procedure, as each oral health indicator is widely used in Japanese clinical and community settings [17]. Oral frailty data regarding mastication, swallowing, oral motor skills, and salivation were collected and assessed in accordance with the operational definition proposed in the latest international e-Delphi study [18]. During the oral examination, participants were instructed to wear removable dentures, if applicable. Mastication was evaluated using a color-changing chewing gum designed to assess mixing ability (XYLITOL; Lotte Co., Japan). The participants were instructed to chew the gum freely for 60 s. After chewing, the gum, which was expected to change color from green to red, was spread onto a thin layer, and color change was measured using a colorimeter (CR-13; Konica-Minolta Co., Tokyo, Japan) to determine the a* value in the CIE L*a*b* color space [19]. Decreased masticatory ability was defined as an a* value of <14.2 for men and <10.8 for women [20]. Swallowing was evaluated using a questionnaire that included the following question: “Do you experience coughing when drinking tea or soup?” [21]. Participants who responded affirmatively were classified as having decreased swallowing function. Oral diadochokinesis (ODK), a common indicator of oral motor function, requires participants to repeat the syllable /ta/ as quickly as possible for 5 s. The number of repetitions per second was recorded using a digital counter (T.K.K.3350; Takei Scientific Instruments Co., Ltd., Niigata, Japan). Reduced articulatory oral motor skills were defined as <6.0 repetitions/s [17]. Salivation was assessed using self-rated oral dryness based on the following question: “Do you often feel that your mouth is dry?” [21]. Oral frailty was defined as the number of deficits in the above four indicators.

Other oral health variables included number of remaining teeth and tongue pressure. The number of remaining teeth was defined as the total number of erupted teeth, excluding residual teeth and teeth with grade 3 mobility [5]. Tongue pressure was measured using a JMS tongue pressure measurement instrument (GC, Tokyo, Japan), and the maximum value from three trials was recorded [22]. Decreased tongue pressure was defined as tongue pressure <30.0 kPa [17].

### 2.3 Intrinsic capacity

The concept of intrinsic capacity was proposed by the World Health Organization (WHO) [2]: five domains—vitality, psychological, cognitive, locomotive, and sensory—were subsequently proposed [23,24]. Vitality was evaluated according to definitions proposed by the WHO [25]. Weight loss, as part of the nutritional status, was evaluated in the energy and metabolism domains, and handgrip strength was evaluated in the neuromuscular function domain. The measurement procedures and cutoff values were adopted from the revised Japanese version of the Cardiovascular Health Study criteria [26]. Weight loss was defined as a loss of ≥2 kg over the past 6 months; low handgrip strength was defined as <28 kg for men and <18 kg for women. Participants with weight loss or low handgrip strength were classified as having low vitality. Psychological and cognitive function were assessed using the Kihon Checklist, a validated screening tool for functional disability in Japan [27]. The cognitive and psychological domains of the checklist correlate with the Mini-Mental State Examination and Geriatric Depression Scale-15 [28]. Regarding cognition, the following three questions were asked: “Do your family or friends point out your memory loss?,” “Do you find yourself not knowing today's date?,” and “Do you make a call by looking up phone numbers?” Participants were classified as having cognitive decline if they answered “yes” to either of the first two questions or “no’’ to the final question. Regarding psychological status, the following five questions were asked: “In the last two weeks, have you felt a lack of fulfilment in your daily life?,” “In the last two weeks, have you felt a lack of joy when doing things you used to enjoy?,” “In the last two weeks, have you felt difficulty in doing what you could easily do before?,” “In the last two weeks, have you felt helpless?,” and “In the last two weeks, have you felt tired for no reason?” Participants were considered to have psychological problems if they responded “yes” to at least one question. Locomotive capacity was evaluated based on gait speed [23]. The participants performed a 4-m walking test; a gait speed of <1.0 m/s was indicative of locomotive deficit [26]. Sensory function was evaluated on the basis of self-reported vision and hearing. Visual impairment was defined as a self-reported diagnosis of cataract or glaucoma based on the following question: “Have you been diagnosed with cataract or glaucoma?” Hearing impairment was assessed using self-rated hearing based on the following question: “Do you suffer from hearing loss?” Participants were classified as having sensory impairment if they answered “yes” to either of the questions. The intrinsic capacity was scored based on the number of deficits in these domains.

### 2.4 Dietary variety and homebound status

Dietary variety was evaluated using the Dietary Variety Score (DVS), a validated measure of dietary diversity among older adults [29]. Participants were asked about their frequency of consumption of 10 food groups (meat, fish, shellfish, eggs, soybean products, milk and dairy products, green and yellow vegetables, other vegetables, fruits, seaweed, oils, and fats). The number of food groups consumed “almost every day” was summed to create the DVS (range, 0–10): higher scores indicate greater dietary variety and better nutritional balance. In this study, a score <7 points was defined as poor dietary variety [29,30]. Homebound status was assessed using the Kihon Checklist question: “Compared with last year, do you go out less frequently?” Participants who answered “yes” were classified as homebound [31].

### 2.5 Covariates

Information regarding age, sex, education, smoking and drinking habits, and comorbidities was collected using a self-administered questionnaire. Education, defined as the age at completion of formal education, was classified as <18 years, ≥18 years, or missing. Smoking status was categorized as smoker (current or former) or non-smoker (never). Drinking status was categorized as drinker (current or former) or non-drinker (never). Comorbidities included stroke, hypertension, diabetes, and cancer.

### 2.6 Statistical analysis

Participant characteristics were described using covariates, oral health indicators, dietary intake, homebound status, and intrinsic capacity. Associations between oral health indicators and intrinsic capacity were examined using modified Poisson regression models to estimate rate ratios (RRs) and 95% confidence intervals (CIs). Covariates included age, sex, education, smoking, drinking, and comorbidities (stroke, hypertension, diabetes, and cancer). Covariates used to address confounding factors were selected based on prior research on the association between oral health and intrinsic capacity [15]. To examine effect modification by DVS or homebound status, interaction terms between oral frailty and DVS and between oral frailty and homebound status were incorporated into the multivariable model. To address missing data for covariates, multiple imputations by chained equations created 20 imputed datasets, with estimates combined using Rubin’s rule. “Missing at random” was assumed for missing values. Multiple imputations were performed using all variables included in the analysis. Statistical analyses were performed using Stata version 18.0 (Stata Corp, College Station, TX, USA), and statistical significance was set at α = 0.05. The following two sensitivity analyses were performed: (1) Oral Frailty 5‐item Checklist (OF‐5) was used as the indicator of oral frailty [20], and (2) a complete case was used to analyze the association between oral health and intrinsic capacity.

## 3 Results

**Fig. 1:**
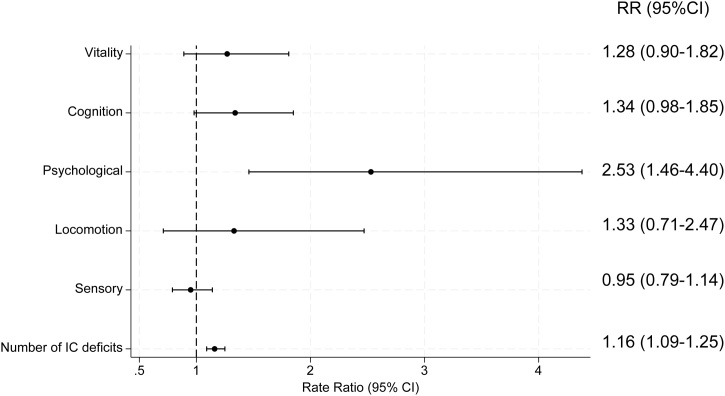
Forest plot showing the association between oral frailty and each domain of intrinsic capacity. Adjusted for age, sex, education, smoking, drinking, and comorbidities (stroke, hypertension, diabetes, and cancer). Abbreviations: RR, rate ratio; CI, confidence interval; IC, intrinsic capacity.

Among the 692 participants, the mean age (standard deviation) was 77.9 years (5.3). Regarding intrinsic capacity, the prevalence of sensory, locomotive, psychological, cognitive, and vitality issues were 41.2%, 7.4%, 11.9%, 22.0%, and 17.3%, respectively (Table 1). The median (interquartile range) number of intrinsic capacity deficits was one (range, 0–2). The prevalence of participants with multiple intrinsic capacity deficits was 27.0%. The prevalence of impaired masticatory ability, swallowing, oral motor function, and oral dryness were 18.6%, 31.5%, 28.2%, and 25.9%, respectively. In the multivariate model, individuals who were older (RR, 1.06; 95% CI, 1.05–1.07) and had more oral frailty deficits (RR, 1.16; 95% CI, 1.08–1.24) were more likely to have intrinsic capacity deficits (operationalized as the number of deficits across five domains), as shown in Table 2. Table 3 shows the associations between oral health indicators and intrinsic capacity. In the multivariate model, individuals with decreased tongue pressure (RR, 1.20, 95% CI, 1.04–1.39), lower ODK (RR, 1.22; 95% CI, 1.05–1.42), impaired swallowing function (RR, 1.25; 95% CI, 1.08–1.46), and oral dryness (RR, 1.32; 95% CI, 1.15–1.53) were more likely to have deficits in intrinsic capacity. Participants with fewer than 20 remaining teeth were not significantly more likely to have intrinsic capacity deficits in the multivariable model (RR, 1.08; 95% CI, 0.94–1.26). Table 4 presents the roles of dietary variety and homebound status in the association between oral frailty and intrinsic capacity using models in which each variable was entered separately into the multivariate model. Analysis showed that the interaction terms between oral frailty and DVS (Model 3: P for interaction = 0.68) and between oral frailty and homebound status (Model 5: P for interaction = 0.71) were not significant. Fig. 1 shows the forest plot of the association between oral frailty and each domain of intrinsic capacity. Oral frailty was found to be significantly associated with psychological status.

**Table 1:** Characteristics of participants aged ≥65 years.

Variables	Total (N = 692)
Age, mean (SD)	77.9 (5.3)
Female, n (%)	555 (80.2)
Education, n (%)	
>12 years	124 (17.9)
≤12	563 (81.4)
Missing	5 (0.7)
Smoking, n (%)	
Current	10 (1.5)
Former	73 (10.6)
Never	609 (88.0)
Missing	0
Drinking, n (%)	
Current	203 (29.3)
Former	92 (13.3)
Never	366 (52.9)
Missing	31 (4.5)
Comorbidities, n (%)	
Stroke	92 (13.3)
Hypertension	226 (32.7)
Diabetes	63 (9.1)
Cancer	79 (11.4)
Deficit of Oral Frailty, median (IQR)	1 (0−1)
Poor masticatory ability, n (%)	129 (18.6)
Tongue pressure (kPa), mean (SD)	33.6 (8.5)
ODK /ta/ (times/second), mean (SD)	6.3 (0.8)
Number of remaining teeth, mean (SD)	18.0 (9.4)
Impaired swallowing function, n (%)	146 (21.1)
Oral dryness, n (%)	179 (25.9)
DVS, median (IQR)	5 (3−7)
Homebound, n (%)	162 (23.4)
Deficit of intrinsic capacity domain, median (IQR)	1 (0−2)
Sensory deficit, n (%)	285 (41.2)
Locomotive deficit, n (%)	51 (7.4)
Psychological deficit, n (%)	82 (11.9)
Cognitive deficit, n (%)	152 (22.0)
Vitality deficit, n (%)	120 (17.3)

DVS, diet variety score; SD, standard deviation; IQR, interquartile range; ODK; Oral diadochokinesis.

**Table 2:** Multivariate modified Poisson regression models showing the association between oral frailty and intrinsic capacity* among Japanese older adults (N = 692).

	Model 1**	Model 2
Variables	RR (95% CI)	RR (95% CI)
Age (continuous)	1.06 (1.05–1.08)	1.06 (1.05–1.07)
Female	1.10 (0.90–1.35)	1.08 (0.87–1.36)
<12 years education	0.97 (0.81–1.17)	0.95 (0.80–1.12)
Current or former smoking	0.82 (0.63–1.05)	0.95 (0.72–1.26)
Current drinking	1.14 (0.97–1.36)	1.01 (0.85–1.20)
Comorbidities		
Stroke	1.09 (0.88–1.36)	1.05 (0.86–1.28)
Hypertension	0.95 (0.82–1.11)	0.92 (0.79–1.06)
Diabetes	1.07 (0.85–1.36)	1.13 (0.89–1.43)
Cancer	1.18 (0.95–1.46)	1.10 (0.91–1.33)
Oral frailty (per one deficit)	1.25 (1.16–1.35)	1.16 (1.08–1.24)

Model 1: Null model.

Model 2: Adjusted for age, sex, education, smoking, drinking, and comorbidities (stroke, hypertension, diabetes, and cancer).

Abbreviations: RR, rate ratio; CI, confidence interval.

* Intrinsic capacity is operationalized as the number of deficits across the five domains.

** Each variable was analyzed separately.

**Table 3:** Multivariate modified Poisson regression models showing the association between oral health indicators and intrinsic capacity* among Japanese older adults (N = 692).

		Model 1	Model 2
Variables	Participants (n)	RR (95% CI)	RR (95% CI)
Poor masticatory ability (<14.2 for men; <10.8 for women)	129	1.03 (0.85–1.25)	0.92 (0.77–1.11)
Low tongue pressure (<30 kPa)	218	1.39 (1.20–1.61)	1.20 (1.04–1.39)
Low oral motor function "ta" (<6.0 times/s)	195	1.40 (1.20–1.64)	1.22 (1.05–1.42)
Poor swallowing function	146	1.36 (1.15–1.60)	1.25 (1.08–1.46)
Oral dryness	179	1.34 (1.15–1.57)	1.32 (1.15–1.53)
Number of remaining teeth (<20)	386	1.26 (1.09–1.47)	1.08 (0.94–1.26)

Model 1: Null model.

Model 2: Adjusted for age, sex, education, smoking, drinking, and comorbidities (stroke, hypertension, diabetes, and cancer).

Each variable was analyzed separately.

Abbreviations: RR, rate ratio; CI, confidence interval.

* Intrinsic capacity is operationalized as the number of deficits across the five domains.

**Table 4:** Multivariate modified Poisson regression models showing the association between oral frailty, dietary variety, homebound status and intrinsic capacity* among Japanese older adults (N = 692).

	Model 1**	Model 2	Model 3	Model 4	Model 5
Variables	RR (95% CI), P-value	RR (95% CI), P-value	RR (95% CI), P-value	RR (95% CI), P-value	RR (95% CI), P-value
Oral frailty	1.16 (1.08–1.24), <.001	1.16 (1.08–1.24), <.001	1.13 (0.98–1.31),0.091	1.13 (1.06–1.21), <.001	1.12 (1.02–1.23), 0.018
DVS (<7)	1.09 (0.92–1.29), 0.331	1.08 (0.92–1.27), 0.342	1.04 (0.82–1.32), 0.746		
Oral frailty × DVS			1.03 (0.88–1.22), 0.683		
Homebound	1.54 (1.32–1.78), <.001	–		1.48 (1.28–1.71), <.001	1.44 (1.16–1.78), 0.001
Oral frailty × Homebound					1.03 (0.90–1.17), 0.713

Model 1: Adjusted for age, sex, education, smoking, drinking, and comorbidities (stroke, hypertension, diabetes, and cancer).

Model 2: Adjusted for Model 1 + DVS.

Model 3: Adjusted for Model 2 + the Interaction term between oral frailty and DVS.

Model 4: Adjusted for Model 1+ Homebound.

Model 5: Adjusted for Model 4 + the Interaction term between oral frailty and Homebound.

Abbreviations: DVS, dietary variety score; CI, confidence interval; RR, rate ratio.

* Intrinsic capacity is operationalized as the number of deficits across the five domains.

** Each variable was analyzed separately.

We conducted two sensitivity analyses. When the oral frailty indicator was changed to OF-5 (Supplemental Table S1), oral frailty was significantly associated with intrinsic capacity deficits (RR, 1.44; 95% CI, 1.25–1.65). In the complete-case analysis (Supplemental Table S2), oral frailty was also significantly associated with a greater number of intrinsic capacity deficits (RR, 1.15; 95% CI, 1.07–1.24).

## 4 Discussion

This cross-sectional study demonstrated that various oral functional indicators were associated with intrinsic capacity among community-dwelling older adults after adjusting for age, sex, education, smoking, drinking, and comorbidities, and that effect modification by dietary variety or homebound status was not detected for the association between oral frailty and intrinsic capacity. The findings of the sensitivity analyses are consistent with those of the primary results.

Previous studies have demonstrated an association between oral health and intrinsic capacity. In cross-sectional studies among older adults in the United States, periodontal status, number of remaining teeth, and self-reported oral health, including dental diseases and dental health behaviors, were associated with domains of intrinsic capacity [[13], [14], [15]]. Another study conducted among patients attending a frailty clinic showed that intrinsic capacity deficits were associated with oral hypofunction; however, the results showed a reverse causality to our hypothesis [16]. In this study, various oral functional indicators, including both objective and self-rated measures, were associated with intrinsic capacity deficits and their domains among community-dwelling older adults.

Potential mechanisms linking oral function to intrinsic capacity include dietary variety and social factors. Regarding dietary variety, oral function, particularly masticatory ability, has been reported to be associated with unhealthy dietary intake and dietary patterns [32,33]. Poor dietary intake and patterns are associated with intrinsic capacity deficits [34,35]. Furthermore, regarding the dietary pathway linking oral frailty to intrinsic capacity domains, masticatory impairment and tongue pressure may restrict protein and energy intake, thereby contributing to decline in skeletal muscle mass and compromising vitality and locomotion capacity, potentially leading to sarcopenia [12,36,37]. Regarding psychosocial factors, oral frailty may cause embarrassment during eating and communication, increasing the likelihood of social withdrawal and homebound status, ultimately leading to social isolation among older adults [38,39]. Social isolation is a potential risk factor for intrinsic capacity deficits, particularly affecting the domains of locomotion, cognition, vitality, and psychological capacity [40].

In this study, cumulative oral functional indicators including mastication, swallowing, oral motor skills, and salivation, which can be collected in both research and clinical settings, were associated with intrinsic capacity deficits. These oral functional components can be improved through dental treatments, such as dental prostheses and oral rehabilitation [41,42]. However, medical conditions may also affect oral function. For example, mastication, swallowing, and oral motor skills are affected by sarcopenia and neuromuscular disorders [43,44]. Moreover, oral dryness may occur as a side effect of medications [45]. The findings of the present study suggest that dental professionals should address oral functional decline as a contributor to intrinsic capacity deficits, beyond the conventional focus on dental diseases, to promote healthy longevity in older adults. Furthermore, to support an integrated approach to maintain intrinsic capacity in older adults, oral health assessment in isolation may be insufficient. Instead, it should be integrated into comprehensive geriatric assessment in collaboration with multidisciplinary health professionals [12]. Clinically, the provision of integrated medical and dental services may benefit older adults [46]; however, future longitudinal and interventional studies are warranted to ascertain these benefits.

Oral health may play a significant role in maintaining functional ability through its impact on the intrinsic capacity of an individual, and better oral health has been shown to contribute to healthy longevity [3,5,20,47]. Furthermore, the association between oral frailty and intrinsic capacity deficits remained consistent regardless of homebound status, suggesting that oral health management might be important for all community-dwelling older adults. Therefore, community- and organization-level involvement in removing barriers to accessing dental services may help maintain intrinsic capacity and, consequently, functional ability, particularly among older adults with impaired oral health [46].

This study has several limitations. First, because this was a cross-sectional study, causal relationships could not be inferred. In addition, intrinsic capacity deficits may hinder access to dental care and impair oral self-care owing to physical and psychological decline, thereby contributing to further deterioration of oral health. However, this study was unable to collect detailed information on the functional abilities of each participant. Second, the operational definition of intrinsic capacity is still evolving, and the sensory information in this study was collected based on self-reported eye diseases and hearing problems. To the best of our knowledge, few studies have examined the association between oral health and visual or hearing function. In the present study, no significant association was observed between oral frailty and sensory function. Furthermore, collecting information on the immune and stress response indicators related to vitality is challenging. The reliance on self-reported data and the absence of suggested measures may have led to misclassification; however, the direction of this potential bias remains unclear. Third, because the data were obtained from municipal health checkups, the participants were likely to be relatively healthier. This selection bias may have limited the generalizability of our findings to a broader population. Therefore, future studies using nationally representative samples are required to further investigate this association. Furthermore, self-reported measures of oral functional indicators, homebound status, and sensory status may have introduced an information bias. If such a misclassification was non-differential, the observed associations would likely be attenuated toward the null.

## 5 Conclusion

This cross-sectional study showed that a cumulative decline in oral function was associated with intrinsic capacity deficits among community-dwelling older Japanese adults and that effect modification by dietary variety or homebound status was not identified. Although intrinsic capacity deficits may be identified using a single oral health indicator, the cumulative deterioration in oral function may further amplify these deficits. However, further longitudinal studies are required to confirm this association.

## Author contributions

**Takamasa Komiyama:** conceptualization, acquisition of data, analysis, and interpretation of data, drafting of the original manuscript, and funding acquisition. **Takashi Ohi**: conceptualization, analysis, and interpretation of data and revision of the draft. **Eiko Sato:** Data acquisition and draft revision **Moe Sato:** Acquisition of data and revision of the draft. **Yoshinori Hattori:** conceptualization, analysis, and interpretation of data and revision of the draft.

## Ethical statement

This study was approved by the Ethics Committee of Tohoku University Graduate School of Dentistry (Approval No. 35745). Written informed consent was obtained from all participants before enrollment.

## Declaration of Generative AI and AI-assisted technologies in the writing process

During the preparation of this study, the authors used ChatGPT (AI language model) to help with grammar, spelling, and sentence structure. The model does not produce any analytical or interpretative content. After using this model, the authors reviewed and edited the content as needed and take full responsibility for the content of the publication.

## Funding sources

This work was supported by JSPS KAKENHI Grant Number JP 23K09476.

## Availability of data and materials

The data are not publicly available owing to privacy and ethical restrictions but may be shared upon reasonable request to the corresponding author, subject to ethical approval.

## Declaration of competing interest

The authors declare that they have no conflicts of interest.
