# Supplementary material for: Oral health, dietary variety, homebound status, and intrinsic capacity among community-dwelling older adults
Source: J Nutr Health Aging. 2026 May 19;30(7):100880. doi: 10.1016/j.jnha.2026.100880 (PMC13213796; doi:10.1016/j.jnha.2026.100880)
Supplement: Supplementary file 1 [file mmc1.docx]

**Supplementary material**

Supplemental Figure 1. Flow-diagram of participants

Study participants

N = 834

Participants aged ≥ 65 who attend survey

(n = 782)

Excluding participants aged less than 65 years (n = 3)

Excluding participants without informed consent (n = 49)

Excluding participants missing information on oral health

(n = 1)

Participants aged ≥ 65 for analysis

(n = 692)

Excluding participants missing information on Intrinsic capacity

(n = 89)

Supplemental Table 1. Multivariate modified Poisson regression models showing the association between oral frailty using OF-5 and intrinsic capacity among Japanese older adults (N= 692)

| Variables | Model 1 | Model 2 | Model 3 |
| --- | --- | --- | --- |
|  | RR (95%CI) | RR (95%CI) | RR (95%CI) |
| Age (continuous) | 1.06 (1.05–1.07) | 1.06 (1.05–1.07) | 1.05 (1.04–1.06) |
| Female | 1.04 (0.83–1.31) | 1.06 (0.84–1.32) | 1.02 (0.82–1.28) |
| <12 years education | 0.96 (0.81–1.13) | 0.95 (0.80–1.12) | 0.99 (0.84–1.16) |
| Current or Former Smoking | 0.95 (0.72–1.26) | 0.95 (0.72–1.25) | 0.98 (0.74–1.29) |
| Current drinking | 1.02 (0.85–1.21) | 1.02 (0.86–1.21) | 1.01 (0.85–1.20) |
| Comorbidities |  |  |  |
| Stroke | 1.03 (0.84–1.26) | 1.03 (0.84–1.26) | 1.00 (0.82–1.22) |
| Hypertension | 0.90 (0.78–1.05) | 0.90 (0.78–1.05) | 0.92 (0.79–1.06) |
| Diabetes | 1.06 (0.84–1.33) | 1.06 (0.84–1.33) | 1.01 (0.80–1.28) |
| Cancer | 1.10 (0.91–1.33) | 1.10 (0.90–1.33) | 1.06 (0.87–1.29) |
| OF5 | 1.44 (1.25–1.65) | 1.44 (1.25–1.65) | 1.40 (1.22–1.60) |
| DVS (<7) |  | 1.08 (0.91–1.27) |  |
| Homebound |  |  | 1.49 (1.29–1.73) |

Model 1: Adjusted for age and sex, education, smoking, drinking, and comorbidities (stroke, hypertension, diabetes, and cancer)

Model 2: Adjusted for age and sex, education, smoking, drinking, comorbidities (stroke, hypertension, diabetes, and cancer), and DVS

Model 3: Adjusted for age and sex, education, smoking, drinking, comorbidities (stroke, hypertension, diabetes, and cancer), and homebound

Abbreviations: RR, rate ratio; CI, confidence interval

Supplemental Table 2. Multivariate modified Poisson regression models showing the association between oral health and intrinsic capacity among Japanese older adults in complete case (N= 658)

| Variables | RR (95%CI) |
| --- | --- |
| Age (continuous) | 1.06 (1.05–1.07) |
| Female | 1.06 (0.84–1.34) |
| <12 years education | 0.95 (0.80–1.13) |
| Current or Former Smoking | 0.96 (0.72–1.28) |
| Current drinking | 1.00 (0.84–1.20) |
| Comorbidities |  |
| Stroke | 1.03 (0.84–1.26) |
| Hypertension | 0.94 (0.80–1.10) |
| Diabetes | 1.13 (0.89–1.44) |
| Cancer | 1.08 (0.87–1.34) |
| Oral frailty (per one deficit) | 1.15 (1.07–1.24) |

Adjusted for age and sex, education, smoking, drinking, and comorbidities (stroke, hypertension, diabetes, and cancer)

Abbreviations: RR, rate ratio; CI, confidence interval
